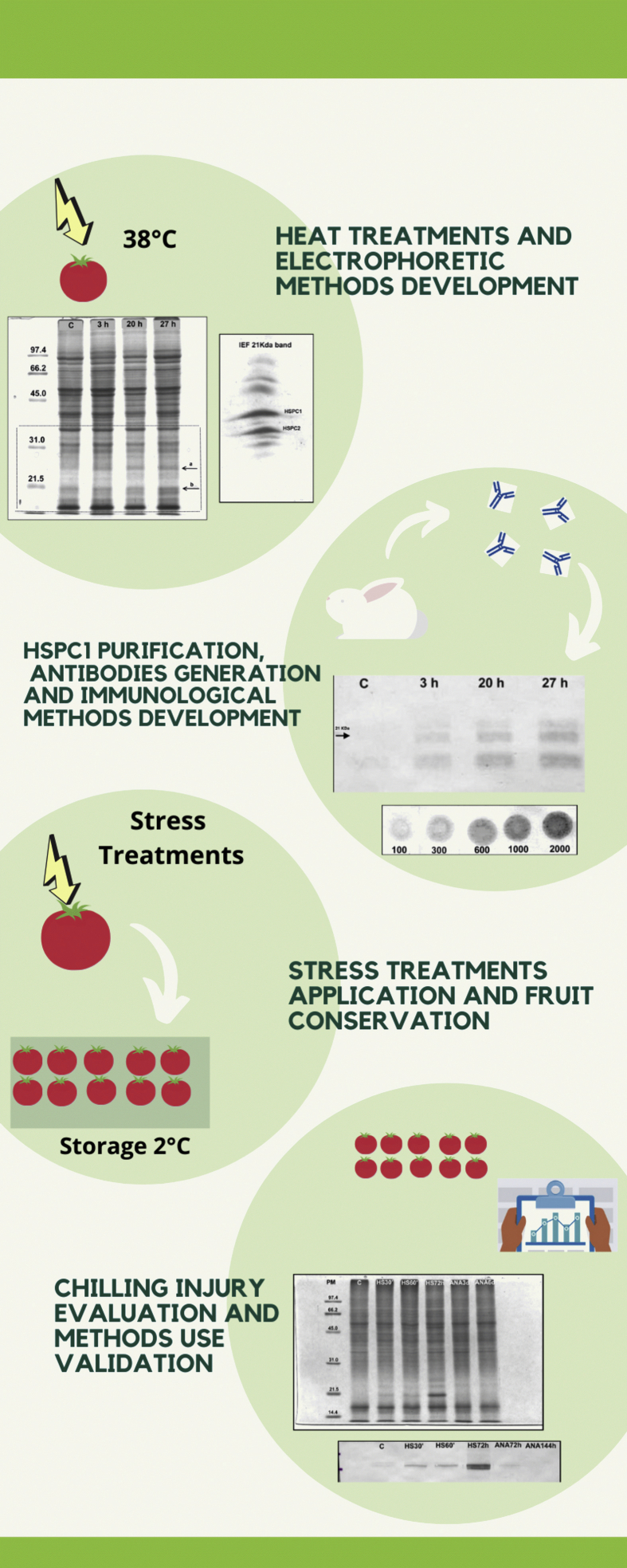# Comparison of different analytical methods to evaluate the heat shock protein (HSP) response in fruits. Application to tomatoes subjected to stress treatments

**DOI:** 10.1016/j.crfs.2020.09.002

**Published:** 2020-10-10

**Authors:** Gustavo A. Polenta, Silvina M. Guidi, Vanina Ambrosi, Gabriela I. Denoya

**Affiliations:** a Instituto Nacional de Tecnología Agropecuaria (INTA), Instituto Tecnología de Alimentos, Argentina; b Facultad de Agronomía y Cs. Agroalimentarias, Universidad de Morón, Morón, Buenos Aires, Argentina; c Consejo Nacional de Investigaciones Científicas y Técnicas, CONICET, Argentina; d Instituto de Biotecnología, Universidad Nacional de Hurlingham (UNAHUR), Argentina

**Keywords:** HSP kinetics, Chilling injury prevention, Heat treatments, Stress monitoring, Dot blot, Immunological methods

## Abstract

Heat shock proteins (HSP) are synthesized in living tissues exposed to transient increase in temperature and play a central role in the protective response against heat and other stresses. In fruits, this response to heat treatment provides resistance to a physiological alteration known as chilling injury. Despite the physiological importance of this group of proteins, publications comparing different methodological alternatives for their analysis are rather scarce. In the present paper, we conducted a comparative study using different electrophoretic and immunological techniques to evaluate the HSP response in fruits. Proteins were extracted from tomato fruit exposed to an HSP-inducing temperature (38 °C) for different times (0, 3, 20, and 27 h). Different alternatives of analysis (SDS-PAGE, SDS-PAGE followed by IEF, Western blot, and dot blot) were performed, and their potential application discussed. The study was complemented with a practical application, in which tomatoes were subjected to heat and anaerobic treatments and then stored in a chill-inducing temperature. This application evidences the relevance of knowing the level of proteins attained by stress treatments which correlates with the acquired tolerance.

## Highlights

• HSP evaluation can be used for practical purposes.
• To assess the HSP response in fruits, different complementary methods should be used.
• A simple method (dot blot) can quantify HSP induced in fruits by heat exposure.
• HSP level induced by stress treatments correlates with acquired physiological tolerance.

## 1 Introduction

It is well known that the exposure of living tissues to a transient temperature rise of 5–10 °C above their normal temperature, induces the synthesis of a specific group of proteins referred to as heat shock proteins (HSPs), which are usually present at low levels in non-exposed cells (Luengwilai et al., 2012). These proteins play a central role in the protective response against heat and other stresses, and in the case of fruits, they are linked to the acquired resistant of heat-treated commodities against chilling injury (Aghdam et al., 2013). From the biochemical point of view, HSP are classified into five different families, according to their molecular masses, each of them having a particular function. The two most relevant families in plants are the 70 kDa family (HSP70) and the small heat shock protein family (sHSP) (Zeng et al., 2016). HSP70 is the most studied group, because of the important function of their members as chaperones. Proteins belonging to this group are involved in relevant processes such as the prevention of protein aggregation, the refolding of denatured proteins, and the translocation of proteins across membranes (Waters, 2013). In turn, the sHSP group constitutes the most diverse group of plant HSP, considering sequence identity, cellular localization, and function. The diversification of this family reflects the evolutionary adaptation to stress conditions unique to plants, such as heat, cold, salinity, oxidative stress, drought, and mechanical injury (Sun et al., 2010). This group shares a common C-terminal sequence of approximately 90 amino acids known as α-crystallin domain (ACD), which is responsible for the reported immunological cross-reactivity among different members (Basha et al., 2012).

The assessment of the presence and over-expression of HSP has also been used with technological purposes. For instance, these proteins can be used to monitor the exposure of living organisms to environmental pollution, since their induction constitutes one of the first detectable biochemical responses against external disturbances, and the increased levels usually persist for periods much longer than other biochemical markers (Basile et al., 2013). In this regard, high concentrations of HSP70 were detected in animals and plants subjected to physical stress or exposed to chemicals such as PCB, DDT, or lindane (Dunlap and Matsumura, 1997). In the field of postharvest technology of fruits and vegetables, HSP constitutes the principal marker to evaluate the level of protection exerted by heat treatments, applied to prevent the development of chilling injury and other physiological and pathological distresses in sensitive commodities. In this regards, different studies were carried out in fruit species such as avocado (Florissen et al., 1996), tomatoes (Ré et al., 2017; Polenta et al., 2007, 2015; Aghdam et al., 2015; Aghdam and Bodbodak, 2014), peaches, plums, bananas, and grapefruits (Aghdam et al., 2013; Aghdam and Bodbodak, 2014).

Despite the growing interest that HSP has raised in plant and postharvest scientists, because of their role in biotic or abiotic stresses, there is a lack of studies comparing diverse alternatives of analysis. In the present paper, we conducted a comparative study of different electrophoretic and immunological analytical alternatives (some of them developed by our group), to detect and evaluate the HSP response in fruits. These techniques were used to assess the biochemical response in tomatoes subjected to different stress treatments, and correlated with the chilling injury protection. The advantages and limitations of each technique are specifically focused and described. This work hypothesizes that for a complete picture of the HSP response, different complementary analyses should be conducted, which can be used as biochemical markers to assess and predict the stress treatment performance in fruits.

## 2 Material and methods

### 2.1 Plant material and treatment application

#### 2.1.1 Model experiment to induce the synthesis of increasing amounts of HSPs, according to the treatment intensity

Mature-green tomatoes (*Lycopersicon esculentum* cv. Cardenal) according to USDA standard (USDA, 1991) of uniform size were obtained from an experimental greenhouse (harvested in October 2015). Fruit were visually selected (60 fruit from an entire lot of 150 fruit, with an average weight of 180 g), and their surfaces were sterilized for 3 min with a chlorine solution (150 mg/kg Cl_2_) at room temperature in a recipient of 100 L, then thoroughly rinsed with tap water in a similar recipient at room temperature for another 3 min, and then left on filter paper to drain.

Thermal treatments were applied by incubation of the fruit in an experimental chamber at 38 °C ± 1 °C and 95 percent relative humidity. Sixty fruit were divided into four lots, and fruit were placed into clean vented plastic trays. Three of these lots were heat-treated for 3 (3 h), 20 (20 h), and 27 h (27 h) respectively, whereas the remaining group received no treatment and was used as a control (C). The experiment was run twice with similar results.

#### 2.1.2 Experiment to assess the HSP response and its correlation with chilling injury (CI) prevention

Nine hundred and sixty mature-green tomatoes (*Lycopersicon esculentum* cv. Colt 45) (USDA, 1991) of uniform size were picked directly from the greenhouse (harvest date: November 2015). Fruits were treated similarly as described in 2.1.1. For the evaluation of the effect of stress on CI prevention, tomatoes were placed into clean vented plastic trays and divided into six lots, each of them submitted to one of the following treatments:

I No treatment, used as control (C).
II Short heat shock treatment (immersion for 30 min in a water bath at 42 ± 1 °C) (HS30′).
III Short heat shock treatment (immersion for 60 min in a water bath at 42 ± 1 °C) (HS60′).
IV Long heat shock treatment (incubation in a traditional chamber at 38 ± 1 °C and 95 percent relative humidity for 72 h) (HS72h).
V Anaerobic treatment (incubation in a 20 L plastic chamber at 20 ± 1 °C, with first a rapid atmosphere exchange by ventilation with humidified nitrogen at a flow rate of 100 ml/min for 2 h, and then a continuous influx of humidified nitrogen at 50 ml/min-flow rate for 3 days) (ANA3d).
VI Anaerobic treatment (incubation in a 20 L plastic chamber at 20 ± 1 °C, with first a rapid atmosphere exchange by ventilation with humidified nitrogen at a flow rate of 100 ml/min for 2 h, and then a continuous influx of humidified nitrogen at 50 cm^3^/min-flow rate for 6 days) (ANA6d).

To evaluate the effect of treatment on the development of CI, fruit were stored for 21 days at 2 °C, and samples were taken under 2 conditions: immediately after treatment and after the storage for 4 additional days in a chamber at 20 °C.

### 2.2 Protein extraction

Proteins were extracted from tomato pericarp following the method of Hurkman and Tanaka (1986) with some modifications. Briefly, fruit were divided into lots of 5 units (individual fruit). Five grams of pericarp were taken from each fruit. The pericarps from these fruit were homogenized in a Waring Blender in liquid nitrogen. The operation was completed by grounding in a mortar, with the addition of liquid nitrogen. One gram from this homogenate was thoroughly mixed in the presence of 1 mL extraction [100 mmol L^−1^ Tris/HCl pH 8.0, containing 1 mmol L^−1^ EDTA, 1 mmol L^−1^ PMSF, and 2% (v/v) β-mercaptoethanol] and 4 mL of phenol saturated with 100 mmol L^−1^ Tris buffer (pH 8.0), and then centrifuged at 21,000×*g* for 10 min at 4 °C. The phenolic phase was recovered, mixed with four volumes of 0.1 mol L^−1^ ammonium acetate (AMA), and incubated overnight at −20 °C. Protein pellets were obtained by centrifugation at 21,000×*g* for 20 min at 0 °C. Pellets were then washed twice with AMA, once with cold acetone (80% v/v), and dried at room temperature. The dried residue was redissolved directly in electrophoretic sample buffer [25 mmol L^−1^ Tris pH 6.8, 1% (w/v) SDS, 10% (v/v) glycerol, 5% (v/v) β-mercaptoethanol, and 0.002% (w/v) bromophenol blue], and boiled for 2 min before being loaded onto a gel and submitted to electrophoresis. Protein concentrations were determined by the Lowry method (Lowry et al., 1951).

### 2.3 Electrophoretic analysis

SDS/PAGE was carried out according to the procedure of Laemmli (1970). For analytical purposes, 15 μg of protein were loaded onto each well of a 0.75 mm-thick gel, whereas for preparative use, 800 μg of protein were loaded onto a 1.5 mm-thick-gel.

Proteins were separated by using 12.5% homogeneous polyacrylamide slab gels. Gels were stained with 0.1% (w/v) CBB solution.

Isoelectric focusing (IEF) was carried out in a vertical system, in a gel composed of 5% polyacrylamide, 0.4% pH 3–10 ampholyte (Pharmalyte, Amersham), 2% pH 4–6.5 ampholyte (Pharmalyte, Amersham), and 8 M urea.

The bands of interest from previous SDS-PAGE analysis were excised, soaked in 20 mmol L^−1^ NaOH for 20 min, and loaded onto the IEF gel. The electrophoresis was run in a Protean II electrophoresis system (BIORAD) at the following voltage steps: 150 V for 30 min, 200 V for 60 min, and 250 V for 90 min. Calibration proteins (Isoelectric point (pI) 4.5–11) were used to estimate the pI of the different protein bands. Gels were stained with 0.1% (w/v) CBB solution. Samples were run in triplicate with similar results.

### 2.4 Antigen preparation and immunization protocol

Protein bands of interest were excised from IEF gels, rinsed several times with Phosphate-buffered saline (PBS) and homogenized in the same buffer. Rabbit immunization for the production of polyclonal antibodies was carried out as described by Polenta et al. (2007). Briefly, rabbits of around 2 kg (3) were injected subcutaneously with 400 mg of HSPC1 excised from IEF gels, and suspended cleaning and sonication directly in 1 mL of PBS buffer emulsified in complete Freund's adjuvant (day 1). Booster injections were administered at days 4 and 14, with the same dose in incomplete Freund's adjuvant. Two or four additional injections were performed and blood samples were withdrawn 1 week after each injection. Animals were maintained under conditions that fulfilled all ethical and scientific requirements for animal use included in EU Directive 2010/63/EU. Pre-immune serum (day 0) was considered as negative control. Antiserum containing the polyclonal antibodies against HSPC1, one of the sHSP, was aliquoted and stored at −80 °C until use.

### 2.5 Immunoblotting

Separated polypeptides were transferred (50 min at 100 V) onto a nitrocellulose membrane (0.45 μm) by using a Mini Protean II Electrophoresis System (BIORAD). In the case of the s HSP, the polyclonal antiserum was raised against HSPC1 (diluted 1:750), which was used as the primary antibody. Anti-rabbit IgG raised in goat and conjugated to alkaline phosphatase (BiORAD, dilution 1:1500) was used as the secondary antibody. In the case of HSP70, a commercial monoclonal antibody (SIGMA, cat H5147, diluted 1:1500) was used as the primary antibody, while anti-mouse IgG raised in goat and conjugated to alkaline phosphatase (BIORAD, dilution 1:1500) was used as the secondary antibody. Membranes were revealed with nitroblue tetrazolium chloride and 5-bromo-4-chloro-3-indolyl phosphate. In each experiment, samples were run by triplicate with similar results.

### 2.6 Dot blot

For the dot blot analysis, 40 μg of total protein was directly deposited with an automatic pipet onto the nitrocellulose membrane (Hybond, Amersham, 0.45 μm pore size). Quantification was carried out by setting up a standard in which known amounts of calibrants were deposited. For sHSP evaluation, HSPC1 (from a previous experiment) electrophoretically purified from tomato (cv. Colt 45) and electroeluted from the gel was used as the calibrant, while in the case of HSP70, it was used a commercial protein purified from bovine brain (SIGMA, cat H9776). The absolute amount of protein was expressed in ng of protein, while the relative amount was referenced to the initial amount present in untreated fruit (considered as 100%). Calibrants were deposited in triplicate, with the values shown in Table 1 representing the average value for each concentration.

**Table 1:** Amount of proteins as quantified by dot blot revealed with antiserum obtained from rabbit immunized against HSPC1, or with a commercial monoclonal antibody (anti-HSP70). Analyses were carried out on 40 μg of total protein, and results are expressed as an absolute amount (ng of protein ± Std error) and amount relative to the initial amount present in untreated fruit (considered as 100%). Pearson correlation coefficient between treatment intensity (time in h) and protein amount was 0.91 (p < 0.01) for the case of sHSP, and 0.95 (p < 0.01) for the case of HSP70. CV: Coefficient of Variation.

	Treatment				
		Control	3 h	20 h	27 h
sHSP	Absolute amount (ng)	645 ± 81	1653 ± 81	2473 ± 303	2768 ± 265
	Amount relative to control (%)	100	256	383	429
	CV (%)	12.8	5.2	19.3	14.7
HSP70	Absolute amount (ng)	899 ± 31	4726 ± 72	7406 ± 232	11,023 ± 374
	Amount relative to control (%)	100	526	824	1226
	CV (%)	5.3	2.1	4.2	4.5

### 2.7 Image analysis

Gels were analyzed with a Bio-Rad GS-800 Imaging Calibrated Densitometer and digitally processed by Quantity One 1-D Analysis software. Lane- and band-based functions were used to determine apparent molecular weights (MWs), pIs, and relative and absolute amounts of proteins. A known amount of Bovine Serum Albumin (BSA) was used as protein standard for lane-based protein quantitation. Samples were quantified by triplicate, with the values shown in Table 1 representing the average value.

### 2.8 Chilling injury evaluation

The establishment of a CI-inducing condition was determined by a storage temperature considerably lower than the reported threshold for the damage (2 °C, threshold temperature: 12.5 °C) and by storage time longer enough to induce the development of symptoms (21 days). Considering that in tomatoes, the main symptoms of CI are the increased rate of fungal infection and the presence of pitting, decay was evaluated visually, as the presence of macroscopic fungal growth, and pitting as the presence of more than one spot. The corresponding percentages of diseased fruit, and fruit with visual pittting were recorded (Efiuvwevwere and Thorne, 1988; El Assi, 2004; Biswas et al., 2016).

## 3 Results

### 3.1 SDS-PAGE analysis

**Fig. 1:**
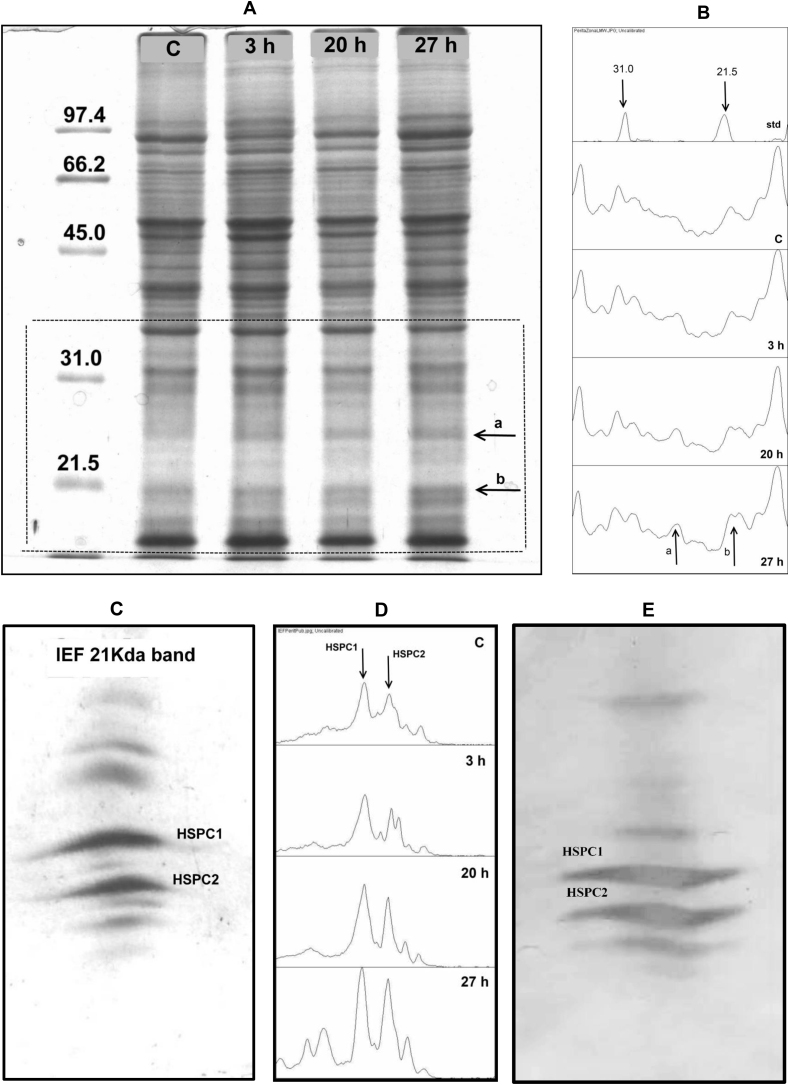
(A) SDS ⁄ PAGE of protein extracts from tomatoes untreated (Control, C), or treated for 3 (3 h), 20 (20 h), or 27 (27 h) hours at 38 °C. (B) Densitometric analysis of the low molecular weight region of the gel (indicated by a dotted line in Fig. 1A). Proteins showing an important increase are indicated by arrows. (C) IEF pattern of the 21 kDa band excised from the SDS ⁄ PAGE shown in Fig. 1A (indicated by arrow b) (D): Densitometric analysis of the IEF gel corresponding to tomatoes untreated (C) or submitted to 38 °C for 3 (3 h), 20 (20 h), or 27 (27 h) hours. The most prominent proteins (termed HSPC1 and HSPC2), which also showed important increases with the duration of treatments, are indicated by arrows. (E) Western blot analysis of the IEF of the 21 kDa band excised from the SDS ⁄ PAGE as shown in (C).

Fig. 1A shows the SDS-PGE analysis of protein extracts from tomatoes exposed to 38 °C for different periods (0, 3, 20, and 27 h). As evidenced, this technique made possible the detection of a prominent group of proteins induced by heat exposure, with molecular masses ranging from 15 to 35 kDa, which is compatible with the sHSP characteristics. Since these proteins are located in a region of the gel with a low density of proteins, the electrophoresis was complemented with densitometric analysis. Therefore, the relative amount of protein induced by each treatment (Fig. 1B) could be estimated, showing that the most significant increase in intensity corresponds to the 21 kDa and 25 kDa protein bands.

### 3.2 SDS-PAGE followed by IEF for the analysis of specific bands of interest

Since 1D electrophoresis cannot resolve individual proteins with similar MW, the 21 kDa protein band was excised from the SDS/PAGE and subsequently separated by IEF (Fig. 1C). This technique resolved the band of fruit heated for 27 h into a set of up to 9 different proteins. It is important to highlight that the increment of sHSP was already detected after 3 h of treatment, which shows that this combined technique constitutes an early and specific monitoring tool. Additionally, this method allows the estimation of the main physicochemical parameters of the individual proteins (isoelectric point and molecular mass). The IEF gel was subjected to densitometric analysis (Fig. 1D), which permitted to estimate the intensity of the bands, each of them representing an individual protein. Therefore, the relative amount of proteins induced by the different treatments could be compared, which shows that the two main proteins, termed HSPC1 and HSPC2, represent, altogether, approximately more than 75% of the small heat shock proteins induced by the treatment. These two proteins, together with most of the proteins present in the original SDS/PAGE band, reacted with the anti-HSPC1 rabbit antiserum (Fig. 1E), which evidences that they belong to the sHSP family.

### 3.3 Western blot analysis

**Fig. 2:**
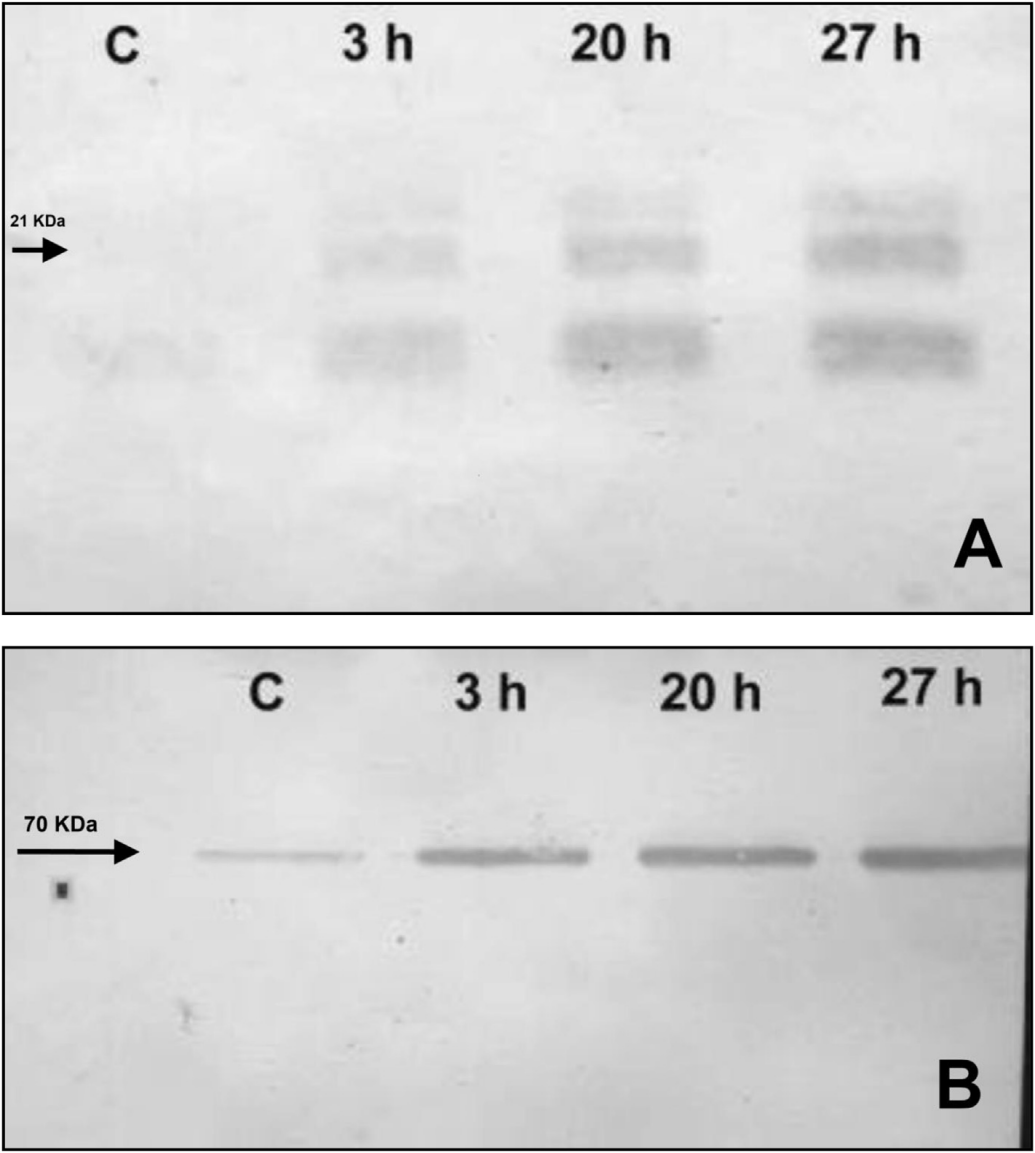
Western blot analysis of protein extracts from tomatoes untreated (Control, C), and treated for 3 (3 h), 20 (20 h), or 27 (27 h) hours at 38 °C. Membranes were revealed with antiserum of rabbit immunized with HSPC1 protein (A), or with commercial monoclonal antibody anti-HSP70 (SIGMA, cat H5147).

Fig. 2 shows Western blot analysis of tomatoes submitted to different intensities of heat treatments (0, 3, 20, and 27 h). Membranes were revealed with two types of antibodies: anti-HSPC1 rabbit antiserum obtained by our group (Fig. 2A), and commercial anti-HSP70 monoclonal antibodies (Fig. 2B). Remarkably, this last antibody, which was raised against a protein from cow brain, recognized the stress proteins induced in tomato. The use of highly specific antibodies provides unambiguous evidence that the over-expressed proteins belong to the two most important HSP families and permitted the analysis of each family.

As shown in Fig. 2B, an important basal level of HSP70 was already present in control fruit, and increased thereafter, proportionally to the treatment intensity. In the case of sHSP, a low basal level was also detected, which increased after heat exposure, according to the treatment intensity (Fig. 2A), and in a pattern similar to the HSP70 family.

### 3.4 Dot blot analysis

**Fig. 3:**
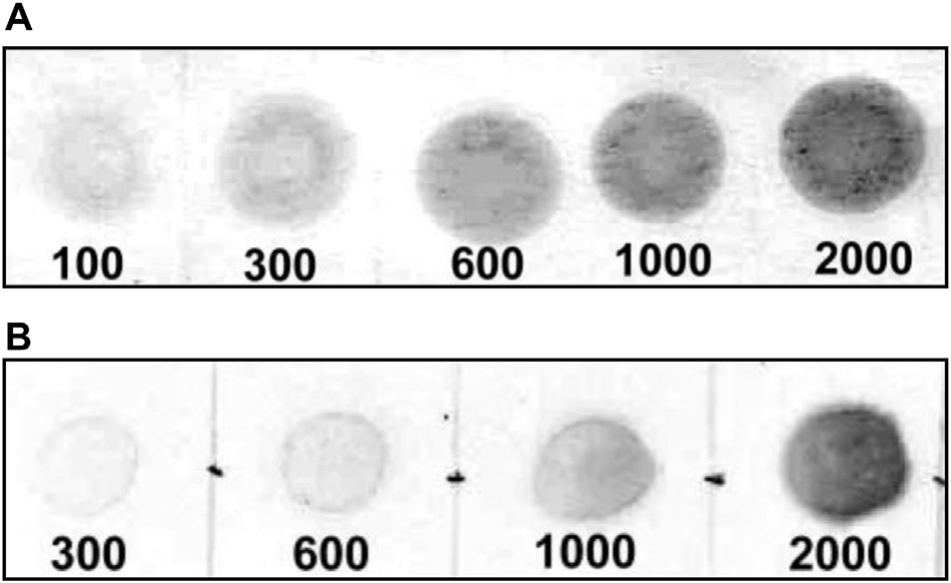
Calibration standard curve used for the quantification of the dot blot analysis. The calibration proteins used s were HSPC1 from a previous experiment, electrophoretically purified from tomato (cv Colt 45), and electroeluted (A) or commercial HSP70 purified from bovine brain (B - SIGMA, cat H9776).

Results show that dot blot offers a simple and accurate way to specifically quantify the amount of HSP induced in fruits by heat exposure. To estimate the absolute amount of proteins, we set up first a calibration curve, by loading different amounts of the target proteins onto a nitrocellulose membrane and revealing them with the immunologic system described in Methods (primary and secondary antibodies, and chromogenic substrate). Fig. 3A shows the standard curve for the sHSP group, which was obtained by using an electrophoretically-purified protein (termed HSPC1), while for the HSP70 family, the calibration curve was obtained with a purified commercially available HSP70 from SIGMA® (Fig. 3B).

The estimated limits of detection for the method were 100 ng and 300 ng for HSPC1 and HSP70 respectively. Calibrations curve were adjusted to a second order polynomial, with an R square of 0,95, and CV among 15 and 21% (depending on the calibrant concentration) for sHSP; and an R square of 0.97 and CV among 12 and 23%, for HSP70.

For the analysis of the samples, protein extracts from the treated tomatoes were diluted, if necessary, until the measured intensity lied within the range of the calibration curve. Table 1 shows the absolute amounts of protein in the treated tomatoes, as calculated in the densitometric analysis of the dots. For quantitation purposes, the images of the membranes were digitalized, and the dots intensities measured with a user-friendly open-source software (ImageJ®). By comparing the intensities of control and treated samples, it was possible to estimate the increase in sHSP concentration, even in tomatoes submitted to the lowest combinations of time-temperature (30 min at 42 °C – data not shown, or 3 h at 38 °C). This table also shows absolute concentrations of sHSP and HSP70, as well as their relative amounts, by reference to the original amount present in control fruit (considered as 100%). In the case of sHSP, coefficients of variation (CV) showed values among 7.6 and 14.6%, for repeatability, and among 8.8 and 18.7% for reproducibility. For the case of HSP70, values were among 4.7 and 9.9% (reproducibility), and among 8.4 and 11.1 (reproducibility). Pearson correlation coefficient between treatment intensity (time in h) and protein amount were 0.91 (p < 0.01) for the case of sHSP, and 0.95 (p < 0.01) for the case of HSP70.

Owing to the universal character of HSP, it is expected that this technique be capable of quantifying the level of HSP attained after the exposure of any plant tissues to heat or other stresses.

### 3.5 Practical application of the methodologies

Through the design of a practical experience, we evaluated the performances of the proposed methods. The experiment involved the application of different stress treatments, the subsequent evaluation of the HSP content, and the link between HSP synthesis and the performance of the treatments to prevent the development of CI. Tomatoes were either untreated or subjected to different intensities of heat or anaerobic treatments, and the most relevant results are presented in the following items.

#### 3.5.1 Physiological evidence of chilling injury

After treated, fruit were stored in a chilling injury-inducing condition (2 °C) for 13 and 21 d, and evaluated immediately after cold withdrawal, and after 4 days at 20 °C, to induce the development of the chilling injury symptoms, as described by Biswas et al. (2016). In fruit evaluated after treatments, or after withdrawal from cold storage, no symptoms of chilling injury were evident (data not shown). Symptoms were evident to different extents only after 4 days at 20 °C, as shown in Table 2.

**Table 2:** sHSP induction and chilling injury symptoms (spoilage and pitting) in tomatoes submitted to the different treatments. The absolute amount of proteins (ng of sHSP included in 40 μg of total protein of the extract ± Std error) as quantified with the antibody obtained by immunizing rabbits with HSPC1 protein and amounts relative to those present in untreated fruit (control), considered as 100%. Percentages of fruit with spoilage or with pitting in tomatoes subjected to the different treatments and stored for 20 days at 2 °C, after 4 days of exposure to 20 °C to induce damage.

Treatments	Absolute amounts of sHSP (ng)	Variation Coefficient (%)	Amounts relative to Control (%)	Immediately After Treatment + 4 days at 20 °C		21 days at 2 °C + 4 days at 20 °C	
				Spoiled Fruit (%)	Fruit w/pitting	Spoiled Fruit (%)	Fruit w/pitting
Control	616 ± 63	14,8	100	0	0	12,5	6,25
HS30′	1545 ± 149	13,9	251	0	0	0	0
HS 60′	1663 ± 110	9,5	270	0	0	6,25	0
HS72hs	4130 ± 300	10,5	670	0	0	100	NE
ANA3D	970 ± 76	11,3	157	0	0	12,5	0
ANA6D	480 ± 60	18,0	78	31,25	12,5	43,75	0

#### 3.5.2 SDS-PAGE analysis

**Fig. 4:**
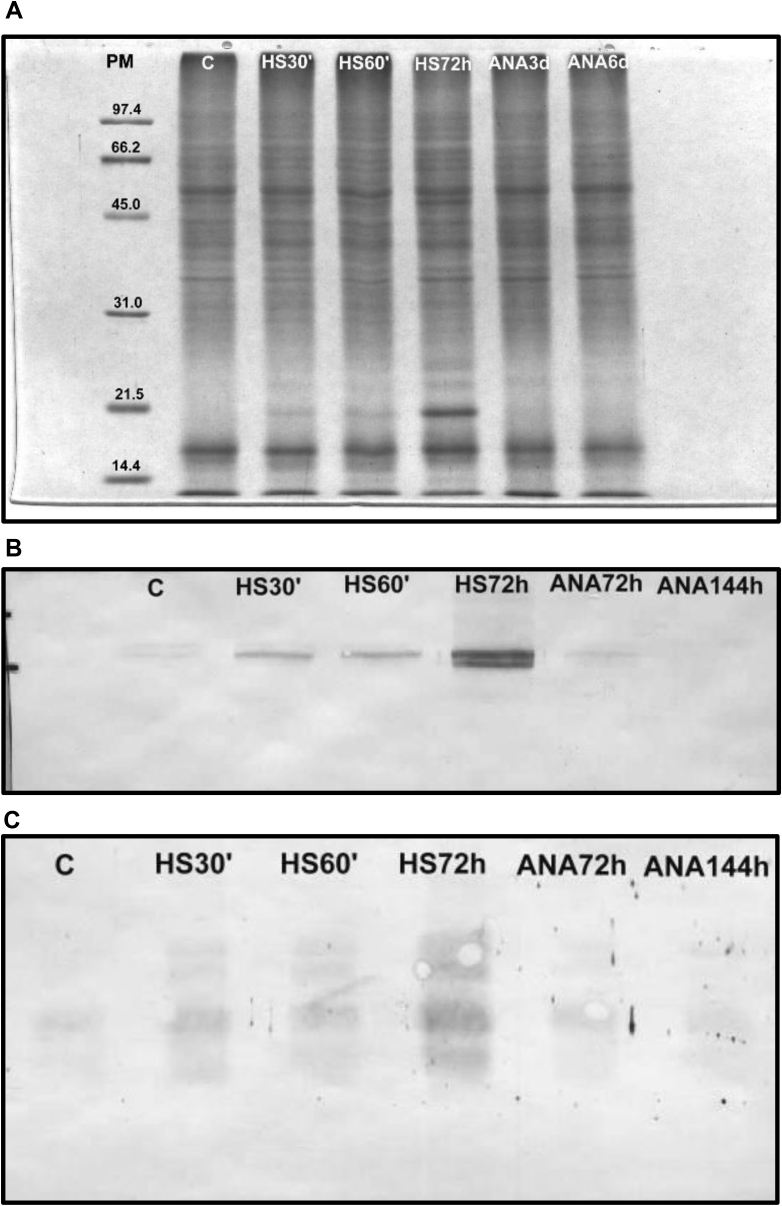
(A): SDS ⁄ PAGE of protein extracts from tomatoes untreated (Control, C), exposed at 38 °C for 30 min (HS30′), 60 min (HS60′), 72 h (HS72h), or at anaerobiosis for 3 (ANA3d) or 6 days (ANA6d) 27 (27 h) hours at 38 °C. Western blot analysis was revealed with antiserum of rabbit immunized with HSPC1 protein (B), or with commercial monoclonal antibody anti-HSP70 (C) SIGMA (cat H5147).

Fig. 4A shows the protein pattern of extracts from tomatoes untreated (Control) or subjected to the different treatments (HS30′, HS60’, HS72h, ANA3d y ANA6d). Samples were analyzed immediately after treatments, and after 21 d of storage at 2 °C. Protein pattern was similar to those described in 3.1, with several new bands in heat-treated fruit, in the region of low MW (as indicated by arrows), the most prominent of them being a band of around 21 kDa. Interestingly, this band became evident immediately after treatment and remained visible during the entire storage at 2 °C (Data not shown). No band with these characteristics was apparent, either in untreated tomatoes or in fruit subjected to anaerobic treatments (ANA3d and ANA6d). Among the different treatments, fruit exposed to heat for 72 h (HSP72h) showed the highest intensity of bands.

In the region of molecular weights around 70 kDa, the high density of proteins in the gel made it difficult to detect differences in the protein patterns among treatments.

#### 3.5.3 Inmunoblots

Fig. 4B shows Western blot analysis revealed with the commercial anti-HSP70 monoclonal antibody. As shown in this figure, members of this family were constitutively expressed in untreated tomatoes, while heat treatments induced the synthesis of additional amounts of proteins, in concentrations correlated with the treatment intensities. Particularly, in tomatoes subjected to the HS72h treatment, additional bands of proteins belonging to the same family were also detected.

Fig. 4C shows Western blot analysis revealed with the anti-HSPC1 rabbit antiserum. It is important to mention that the protein used to generate the antibodies in rabbits was induced in this experiment by the exposure of tomatoes for 72 h at 39 °C (HSP72h treatment). This treatment caused the most remarkable overexpression of HSP, in general, and of sHSP, in particular. As shown in this figure, a basal level of sHSP was already present in untreated fruit, although at a very low concentration.

Table 2 presents the amounts of HSP induced by each treatment, estimated by using the purified HSPC1 as a quantitative reference (absolute amount), or referred to those present in untreated fruit, considered as 100% (relative amount). Interestingly, short heat treatments (HS30′ y HS60’) increased the initial amount of protein by approximately 2.5 times, while in the longest heat treatment (HSP72h), the increase was approximately 6.7 times. In turn, the anaerobic treatments had no effect on sHSP synthesis, indeed provoking a slight decrease in their concentration. This fact can be also appreciated in SDS-PAGE and Western blot analyses (Fig. 4).

## 4 Discussion

### 4.1 Assessment of the techniques

The four techniques evaluated in the study were capable of detecting and/or quantifying the increase in HSP in a model experiment, in which different treatment intensities were used. The techniques showed their capability to assess the kinetics of HSP synthesis and give a complete picture of the HSP response, and can be used independently, or as a set of analyses, since there are complementary each other. This capability is qualitatively shown, in the case of the electrophoretic and Western blot analyses, and quantitatively, in the case of dot blot. This information is valuable from the technological point of view, considering that, as shown in previous studies, the amount of the induced proteins properly reflects the level of stress undergone by tissues. Among the different studies on this subject, the level of overexpression of HSP were used to evaluate and monitor the optimal protection induced by stress treatments in chilling sensible commodities such as tomatoes (Polenta et al., 2015), citrus (Polenta et al., 2007), banana (He et al., 2012) and avocado (Kassim et al., 2013). In this last commodity, Florissen et al. (1996), correlated the minimum time required to induce the synthesis of HSP with the performance of the treatment. Interestingly, the ability of living organisms, including plants, bacteria, and animals, to withstand high temperatures can be correlated with their capacity to accumulate HSP (Sung et al., 2014).

For individual use, the selection of each technique will depend on aspects such as the levels of detail required, the feasibility of application of each method, the equipment and reagents available (especially immunosera), and the particular objectives of the research.

Despite its simplicity, the combination of SDS-PAGE with densitometry provides precise information on MWs of the induced proteins, and also permits the semi-quantitation (estimation) of HSP accumulation. However, in regions with a high protein density such as the 70 kDa region, it is difficult to properly identify the protein/s of interest and, therefore, detect small variations, for which more complex immunological techniques would be necessary.

The combination of SDS-PAGE + IEF offers additional information, such as MW and pI of individual proteins, but the type of HSP analyzed is rather limited to specific regions represented, in this particular case, by the lower MW range of the sHSP family. The proposed modification of the 2D-IEF-SDS/PAGE protocol, in which the classical steps were inverted, had two main positive effects: first, the resolution of the protein isoforms was improved, since IEF offers a considerably better-resolving performance than SDS-PAGE. This is because IEF can concentrate, within each gel band, the protein molecules, while in the latter, protein molecules tend to diffuse as the electrophoresis progresses. Therefore, this advantage would have been lost if IEF had been used as the initial step. In addition, the total amount of protein loaded onto the gels could be greatly increased (800 μg of total protein), a feature that makes this method also suitable for preparative purposes (i.e. protein purification to generate antisera). In fact, the method was used, in the present research, to purify and use one of the sHSP (HSPC1), which was used as a calibrant for the standard curve in the dot blot technique (Fig. 3A and Table 1).

From the point of view of the method sensitivity, the overexpression of proteins induced by the treatments could be easily detected within 3 h of heat exposure, while longer treatments rendered concentrations increasingly higher. Interestingly, no apparent maximum was attained in the present research, even after 27 h of heat exposure, which is in contrast with some previous studies, where a plateau in HSP concentration was attained after a few hours. Among these investigations, a rapid increase in HSP concentration was verified in rice leaves within the first 2 h of exposure to high temperature (Lee et al., 2013). Another study reported that HSP70 increased gradually, although was especially abundant from 2 h to 24 h after heat stress (Miova et al., 2015).

The high number of different proteins belonging to the sHSP family evidences the complexity of the heat shock response, and is comparable with previous studies. In this regard, it was reported that *Arabidopsis thaliana* can accumulate up to 19 new proteins, with estimated molecular masses between 15 and 25 kDa (Santhanagopalan et al., 2015). In protein extracts from heated tomato cells, three 20-kDa HSP with pIs ranging from 7.0 to 7.3, and five 21-kDa proteins with pIs between 5.1 and 6.0 were isolated (Nover and Scharf, 1984).

When more detailed and specific information is required, Western blot analysis has the advantage of combining the specificity and sensitivity of immunological methods, with the advantage of the resolution associated with electrophoretic techniques. In this study, this technique permitted the detection of differences in both HSP70s and sHSP accumulation in fruit submitted to different time exposures. As shown in Fig. 2B, control fruit has basal levels of HSP70, which were notably increased after heat exposure, in amounts proportional to the treatment intensity. These basal levels probably correspond to constitutive isoforms of HSP70 (also known as Heat Shock Cognate – Yang and Tohda, 2018), while the augmented amounts detected following heat treatments represent inducible proteins.

In the case of sHSP, the continuous increment evidenced by Western blot was consistent with that observed in the SDS-PAGE analysis. When applying heat treatment with protective purposes, it is important to consider the half-life of the proteins, which was estimated to be approximately 38 h (Puigderrajols et al., 2002).

Another method presented in this study, dot blot, proved adequate for the analysis of HSP, considering its simplicity, specificity, and sensitivity, although its main limitation is the lack of specific information on individual proteins, since no separation step is included. This method can be adapted for use even in small laboratories, since no sophisticated equipment is required.

Table 1 shows the performance of this technique to determine absolute and relative amounts of HSP in tomatoes submitted to treatments of different intensity. Interestingly, the basal amount of sHSP in the variety assayed in this study was similar to that measured by our group in other tomato varieties (unpublished results). It remains to be determined whether this finding can be extrapolated to other species and varieties, which would be helpful to standardize the application of heat treatments. Quantitative data obtained by this method can be employed with predictive and optimizing purposes, to develop mathematical models of HSP induction, as a function of time and temperature exposure, which would be helpful for the successful application of heat treatments in fruits.

Although heat treatment constitutes a promising technology to prevent the development of chilling injury in sensible fruits and vegetables, there are still some technical difficulties preventing its more extensive commercial application (Aghdam et al., 2013). One of them is the narrow range of treatment intensity that separates a successful treatment from a deleterious one (Polenta et al., 2006). Since the level of HSP properly reflects the treatment intensity, we believe that this can be a suitable parameter to implement process control strategies during the treatment application. Other aspects leading to the successful application, such as the treatment uniformity, have been also focused on other studies (Lu et al., 2010). It is expected that, by adjusting these and other parameters, heat treatments could become a widespread technique in the future.

### 4.2 Effect of treatments on the development of chilling injury

The second part of the study was designed to validate the biochemical findings with a practical experience, by using the developed method to assess the HSP profile in fruit submitted to different stress treatments, applied to prevent chilling injury. To stimulate the development of the latent damage induced during storage, fruits were exposed, after cold withdrawal, for 4 days at 20 °C (Aghdam et al., 2014). Results show that, immediately after treatments, only fruit subjected to anaerobiosis for 6 days (ANA6d) had symptoms of physiological damage, even before storage.

In turn, the storage of untreated tomatoes (Control) caused the appearance of visible damage after 21 days. However, as also shown in previous studies (Wang et al., 2015a) the application of short heat treatments (HS30′ y HS60’) prior to storage, decreased the extent of damage, with fruit showing lower percentages of both pitting and decay (Table 2). The beneficial effect of heat treatments and the consequent HSP synthesis was previously shown in different investigations on tomatoes (Ré et al., 2017; Luengwilai et al., 2012; Neta-Sharir et al., 2005). Results show that the effectiveness of treatments was highly dependent on their application at an adequate intensity, since short treatments were much more effective than long treatments, in spite of the higher concentration of HSP attained. These results suggest that mechanisms other than HSP are also involved in stress protection, evidencing an optimal range of intensity that is effective to prevent the development of CI, with treatments beyond this region having a deleterious effect (Aghdam et al., 2015). The development of effective monitoring systems is of utmost importance for the successful application of this technology.

### 4.3 Practical implications of HSP analysis

The present study shows that SDS-PAGE + image analysis permits a simple estimation of the level of sHSP induced by heat treatments, which can accurately reflect the intensity of exposure. Therefore, it constitutes a useful tool for monitoring the induction and continuity of the protecting effect of treatment during storage.

In turn, Western blot constitutes a useful and highly specific tool for monitoring purposes. Proteins belonging to the HSP70 family, in particular, could be considered as a universal tool to assess different stress conditions such as heat, drought, cold, chemicals, and oxidants or pathogens, because of their evolutionary conservation (Ferradini et al., 2015).

In the present research, HSP70 accumulation in treated tomatoes showed the relationship between treatment intensity and protein concentration. Indeed, after exposing the fruit to 72 h at 39 °C, new proteins belonging to this family were detected. Interestingly, anaerobic treatments were not able to induce the synthesis of HSP70, indeed provoking the disappearance of some of the bands present in control samples. Evidently, the biochemical mechanism associated with exposure to anaerobic stress, which proved successful in other studies (Wang et al., 2015b) is different from heat stress, and does not involve the synthesis of HSP70.

Regarding the sHSP group, HSPC1 antibodies had a significant cross-reactivity with other members of this family (Fig. 2, Fig. 4C). This fact was also observed in other species such as rice (Chen et al., 2014). Similarly to the HSP70 family, the level of sHSP accumulation under heat stress depends on the temperature and the duration of the exposure (Yang et al., 2014).

The present research highlights the relevance and practical applicability of the simultaneous detection of HSP70 and sHSP, which are the most relevant HSP families in plants, taking into account their cooperative role in the reestablishment of the cellular homeostasis. In this regard, although studies on HSP have been traditionally carried out separately, more studies focus on the synergistic action of different HSP families (Hasanuzzaman et al., 2013). This universal mechanism of protein protection by HSP is widely distributed among different prokaryotic and eukaryotic species and, therefore, the analysis of these proteins is expected to become increasingly important in any study on stress physiology and stress-based technologies such as chilling injury prevention.

## 5 Conclusions

HSP can be analyzed by different complementary analyses, since these proteins are meaningful markers to optimize the application stress treatments in fruits. Techniques included in the present investigation proved, to different extents, suitable for the identification, estimation, and quantitation of the HSP70 and sHSP groups, which are the most relevant HSP families in plants. The feasibility of the application of each method will strongly depend on the availability of equipment and specific reagents (*ie*. PAGE, Western blot, and IEF equipment and accessories, immunosera, etc.), as well as on the particular objectives of the research. Although each technique has particular advantages and limitations, they are effective to provide relevant information, which can be used for scientific or technical purposes. Although this particular investigation was undertaken in tomato fruit, it can be extended, with minor modifications, to different plant species and tissues, especially for studies dealing with stress physiology. Research in this way is currently underway in our lab.

## Compliance with ethics requirements

All institutional and national guidelines for the care and use of laboratory animals were followed. Animals were maintained under conditions that fulfilled all ethical and scientific requirements for animal use included in EU Directive 2010/63/EU for animal experiments.

## CRediT authorship contribution statement

**Gustavo A. Polenta:** Conceptualization, Investigation, Methodology, Project administration, Writing - original draft, Writing - review & editing. **Silvina M. Guidi:** Investigation, Methodology, Data curation, Writing - review & editing. **Vanina Ambrosi:** Investigation, Methodology, Writing - review & editing. **Gabriela I. Denoya:** Investigation, Methodology, Visualization, Writing - original draft, Writing - review & editing.

## Declaration of competing interest

The authors declare that they have no known competing financial interests or personal relationships that could have appeared to influence the work reported in this paper.

## Figures and Tables

**figs1:**